# Real-world evidence of switching P2Y12 receptor–inhibiting therapies to prasugrel after PCI in patients with ACS: results from EFF-K registry

**DOI:** 10.1186/s12872-022-03034-5

**Published:** 2023-01-09

**Authors:** Jeehoon Kang, Jung-Kyu Han, Han-Mo Yang, Kyung Woo Park, Hyun-Jae Kang, Bon-Kwon Koo, Eun Ho Choo, Jong-Young Lee, Sang-Don Park, Young-Hyo Lim, Hyung-Min Kim, Ji-Hyun Heo, Hyo-Soo Kim

**Affiliations:** 1 Cardiovascular Center, Seoul National University College of Medicine, Seoul National University Hospital, 101 Daehak-Ro Jongno-Gu, Seoul, 03080 Republic of Korea; 2 Department of Critical Care Medicine, Seoul National University Hospital, Seoul, Republic of Korea; 3 Department of Internal Medicine, Seoul St. Mary’s Hospital, Seoul, Republic of Korea; 4 Department of Internal Medicine, Kangbuk Samsung Hospital, Sungkyunkwan University School of Medicine, Seoul, Republic of Korea; 5 Department of Internal Medicine, Inha University Hospital, Incheon, Republic of Korea; 6 Department of Internal Medicine, Hanyang University College of Medicine, Seoul, Republic of Korea; 7 Daiichi Sankyo Korea Co., Ltd., Seoul, Republic of Korea

**Keywords:** Acute coronary syndrome, Prasugrel, Percutaneous coronary intervention, Observational study

## Abstract

**Background:**

Potent P2Y_12_ inhibitors are recommended for up to 12 months after percutaneous coronary intervention (PCI) in patients diagnosed with acute coronary syndrome (ACS). However, the prescription pattern is diverse in real world practice, which includes various switching between antiplatelet regimens. In this study, we analyzed the prescription patterns of prasugrel, and assessed the safety and effectiveness of P2Y12 inhibitors switching patterns in a real world registry of patients subjected to PCI after ACS.

**Methods:**

The EFF-K study included 3077 ACS patients receiving prasugrel-based dual antiplatelet therapy. The cohort was divided into those who were administered with prasugrel as the primary antiplatelet treatment (naïve cohort) or as a substitute agent after clopidogrel or ticagrelor pre-treatment (switch cohort). The primary endpoint was a net adverse clinical event (NACE; a composite of cardiovascular death, non-fatal myocardial infarction, non-fatal stroke, or TIMI major bleeding unrelated to coronary-artery bypass grafting).

**Results:**

A total of 3077 patients diagnosed with ACS were included in the analysis. Among the total population, 726 patients (23.6%) were classed as the naïve cohort and 2351 patients (76.4%) as the switch cohort. Baseline characteristics showed that the switch cohort had more comorbidities, such as hypertension, diabetes mellitus, heart failure and previous PCI. The major cause of switching to prasugrel in the switch cohort was the necessity for a more potent antiplatelet agent (56.3%). During a 12-month follow-up period, 51 patients (1.7%) experienced at least one NACE. The incidence of NACE did not differ between the naïve and switch cohort (1.5% vs. 1.7%, Hazard ratio 1.17, 95% Confidence interval 0.56–2.43, *P* = 0.677). In subgroup analysis, no significant interaction was observed between the treatment strategy and the incidence of NACE across various subgroups.

**Conclusions:**

Dual antiplatelet therapy with prasugrel seems to be safe and effective both as a primary treatment and as a substitute for other P2Y12 inhibitors in a real world registry of Asian ACS patients receiving PCI.

*Trial registration*: KCT0002356, registered June 13, 2017.

**Supplementary Information:**

The online version contains supplementary material available at 10.1186/s12872-022-03034-5.

## Background

Acute coronary syndrome (ACS), in the form of myocardial infarction (MI) with or without ST-segment elevation and unstable angina, remains a major cause of premature death in developed countries [1]. The therapy of ACS is aimed at myocardial reperfusion, primarily through percutaneous coronary intervention (PCI) [2]. While timely PCI is a life-saving procedure, it also poses some risks. Specifically, the intervention disrupts the coronary endothelium, leading to direct exposure of the subendothelium. As a consequence, intracoronary thrombosis may occur during PCI or shortly thereafter. In addition, metal stents, acting as procoagulants, can trigger thrombus formation [3].

Dual antiplatelet therapy (DAPT; aspirin plus a P2Y_12_ inhibitor) lasting for up to 12 months is the treatment strategy recommended by most guidelines for the prevention of atherothrombotic events in patients diagnosed with ACS and undergoing PCI [4]. The potent P2Y_12_ inhibitors produce a stronger antithrombotic effect than their predecessor, clopidogrel, but also pose a higher risk of bleeding, especially when administered chronically. Prasugrel is a third-generation potent thienopyridine that irreversibly binds to the platelet P2Y_12_ receptor and inhibits adenosine diphosphate-induced platelet aggregation. A pivotal study (TRITON-TIMI 38) demonstrated that DAPT with prasugrel was associated with a significantly reduced rate of ischemic events but with an increased risk of major bleeding [5]. Given the intrinsic bleeding risk of antiplatelet agents and distinct effectiveness/risk profiles of various P2Y_12_ inhibitors, prescription patterns tend to be complex, with switching between DAPT regimens depending on the clinical scenario [6]. However, neither previous clinical trials nor guidelines elaborated on how to switch the therapies in real-world practice.

The problem mentioned above seems to be particularly important in East Asian patients, who were shown to be more prone to bleeding and less prone to thrombosis, a phenomenon referred to as the East Asian paradox [7–12]. Indeed, the evidence from some studies suggests that East Asian patients may not benefit from DAPT with potent P2Y_12_ inhibitors equally to other populations, mainly due to higher bleeding event rates [9–13]. However, in a recent phase IV post-marketing surveillance (PMS) study of Korean patients receiving a standard dose of prasugrel, the efficacy and safety of the agent seemed to be similar as in the pivotal trial [14].

The aim of this real-world study was to analyze the prescription patterns of prasugrel, with a particular emphasis on switching between P2Y_12_ inhibitors, and to assess the safety and effectiveness of prasugrel in Korean patients subjected to PCI after ACS.

## Methods

### Study design and patients

**Fig. 1:**
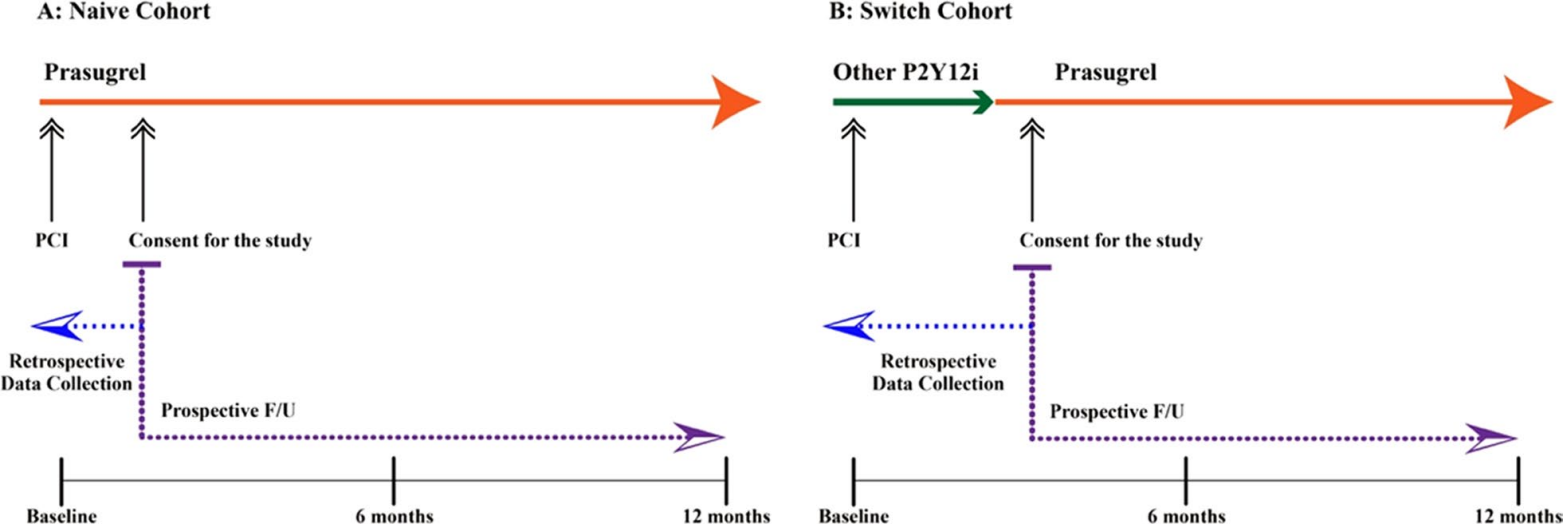
Study scheme

The EFF-K study was a non-interventional, prospective, one-year follow-up cohort research conducted in 52 hospitals located in various regions of South Korea from March 2017 till November 2019. To avoid a selection bias, medical charts of all patients who had been on prasugrel treatment within six months after PCI were sequentially screened for the study. We included only adult patients (≥ 19 years of age) who had been on prasugrel treatment less than six months since PCI and excluded those participating in any interventional study using antiplatelet or anticoagulant agents. Only the candidate patients who voluntarily provided their written consent were registered in the study. The investigators assessed baseline parameters in index PCI. The naïve cohort was defined as those who started receiving prasugrel in the absence of other P2Y_12_ inhibitor(s) before and after PCI. Patients whose antiplatelet medication had been changed from other P2Y_12_ inhibitor(s) to prasugrel within six months since index PCI were defined as the switch cohort (Fig. 1). For the switch cohort, reasons for switching to prasugrel were restricted to the following: adverse events or over-inhibition of platelet aggregation by the previous agent, drug interaction between the previous agent and other concomitant medications, the necessity of a more potent antiplatelet agent, or decreased medication compliance with a twice-daily regimen. Prasugrel regimen, duration of the treatment, and all medical procedures followed the routine clinical practices. The study protocol, including the consent form, was reviewed and approved by the IRB of each institution. The study information was registered in the public domain, the Clinical Research Information Service (https://cris.nih.go.kr/) under the registration number KCT0002356 (13/06/2017).

### Study endpoints and data collection

The primary endpoint of the study was a net adverse clinical event (NACE), defined as a composite of net clinical events, such as cardiovascular death, non-fatal MI, non-fatal stroke, or Thrombolysis in Myocardial Infarction (TIMI) major bleeding unrelated to coronary-artery bypass grafting (CABG). The list of key secondary endpoints included a composite of cardiovascular death, non-fatal MI, and non-fatal stroke as the effectiveness endpoint and a composite of TIMI major or minor bleeding unrelated to CABG as the safety endpoint. Other secondary effectiveness endpoints were individual components, such as cardiovascular death, all-cause death, non-fatal MI, non-fatal stroke, TIMI major bleeding unrelated to CABG, TIMI minor bleeding unrelated to CABG, and urgent target vessel revascularization. Also, serious adverse events and adverse events causing the withdrawal of prasugrel were analyzed as safety endpoints. A serious adverse reaction was defined as one that required hospitalization or prolongation of existing hospitalization, caused congenital malformation, resulted in persistent or significant disability or incapacity, was life-threatening, or resulted in death. Data was collected from the date of registration (i.e. at the time of obtaining an informed consent form) to 12 months after PCI. Although data documentation time points were planned at 1 month (± 2 weeks), 3 months (± 4 weeks), 6 months (± 4 weeks), 9 months (± 4 weeks), and 12 months (± 4 weeks) after PCI, the data were collected even if unplanned follow-up visit occurred during the observational time. The follow-up by telephone calls was not conduced. Electronically captured data, as well as on-site study documents, including signed consent forms, were verified through centralized monitoring and on-site monitoring.

### Statistical analysis

The target number of patients was based on the primary endpoint. Assuming the true incidence of net clinical events of 12.3% based on the TRITON-TIMI 38 study [5], a sample size of 3213 patients was required with adjustment for 15% drop-out rate to achieve the desired precision (target width of 0.025 for confidence interval by Clopper–Pearson method) at 95% confidence level. Continuous variables are presented as mean ± standard deviation (SD) or median (interquartile range [IQR]) as appropriate. Categorical variables are presented as frequency (percentage). As for the effectiveness endpoints, comparisons between cohorts or subgroups were carried out using Pearson’s chi-squared test or Fisher’s exact test depending on the data distribution. Univariate Cox proportional hazards regression was performed to identify independent predictors of NACE and the multivariable model was built with candidate variables being selected if of clinical interest and/or satisfying the entry criterion of *P* < 0.1 in the univariate analysis. Variables included in the model for were carefully selected to avoid overfitting and included old age (≥ 75 years old), sex, hypertension, diabetes mellitus, current smoking, presentation with STEMI, and presence of multivessel coronary disease in angiography. Results are reported as hazard ratios (HR) with 95% confidence intervals (CI). Kaplan–Meier curves were used to assess the incidence and timing of NACE. Adverse events were coded according to the Medical Dictionary for Regulatory Activities (MedDRA), version 22.0. For all analyses, *P* < 0.05 was considered significant. All statistical analyses were conducted using SAS® (version 9.4, SAS Institute Inc. Cary, NC, USA).

## Results

### Patients characteristics

**Fig. 2:**
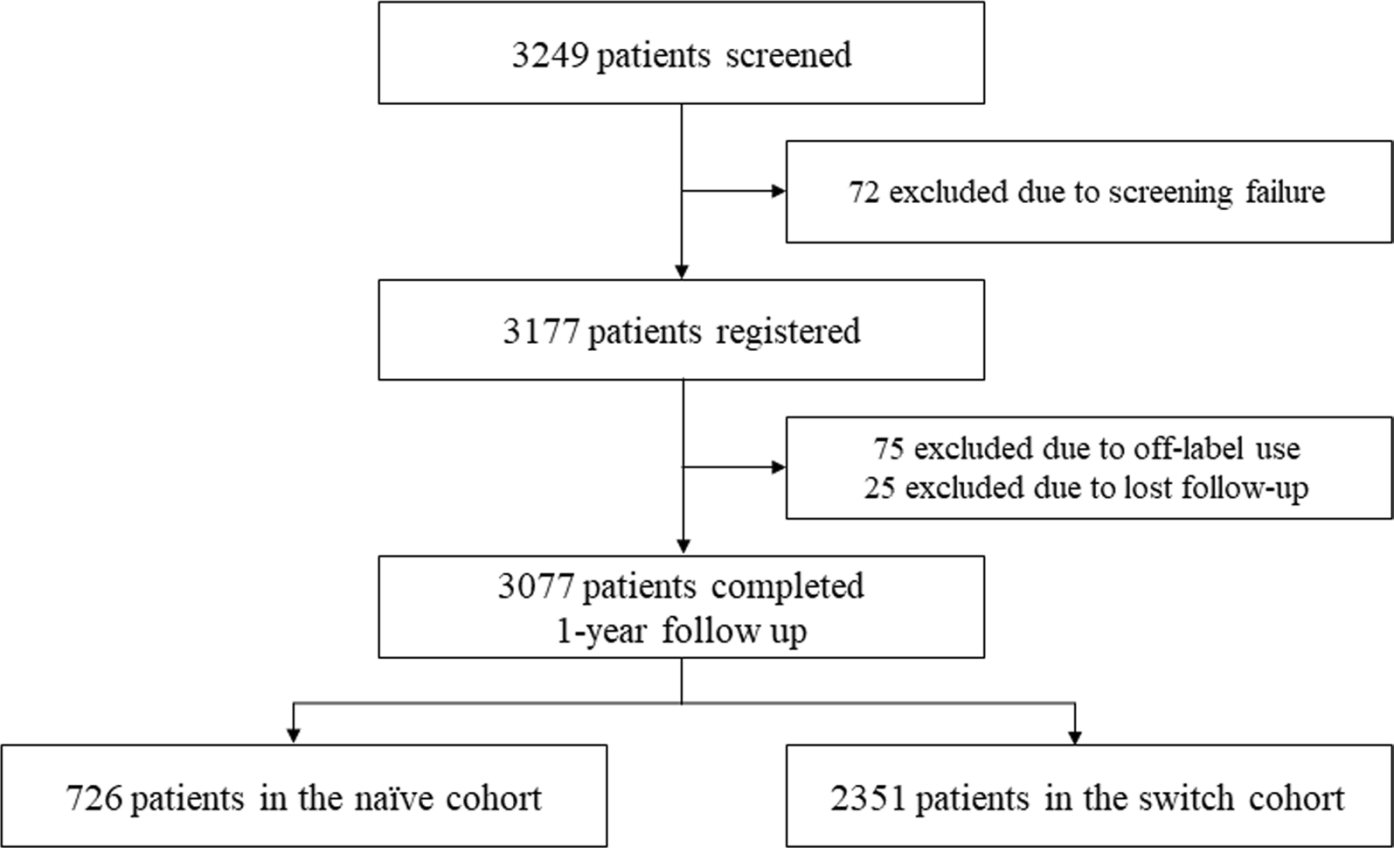
Patient distribution

Among a total of 3249 patients registered in the study, 3077 were included in the analysis. The distribution of patients and the reasons for the exclusion of 172 patients are provided in Fig. 2. About one-fourth of patients (N = 726, 23.6%) were identified as the naïve cohort, and 2351 (76.4%) patients constituted the switch cohort. Patient demographics and baseline disease information are provided in Table 1. The mean age of the patients was 60.6 ± 10.2 years; 776 (25.2%) patients were diagnosed with ST-segment elevation MI at index PCI period. Compared with the naïve cohort, the switch cohort was older, with a higher proportion of female patients. Comorbidities, such as hypertension (naïve vs. switch; 46.0% vs. 55.5%, *P* < 0.001), dyslipidemia (25.5% vs. 36.9%, *P* < 0.001), diabetes mellitus (25.1% vs. 30.8%, *P* = 0.003), heart failure (1.0% vs. 2.7, *P* = 0.007), and previous PCI (5.5% vs. 12.5, *P* < 0.001), were more common in the switch cohort. Also, the proportions of high-risk procedural factors, such as multi-vessel disease (44.7% vs. 53.5%, *P* = 0.001) and ACC/AHA type C lesions (26.4% vs. 51.3%, *P* < 0.001), were higher in the switch cohort than in the naïve cohort.

**Table 1:** Patient Demographics and Baseline Information

	Total (N = 3077)	Naïve cohort (N = 726)	Switch cohort (N = 2351)	P value
Age, years	60.6 ± 10.2	57.7 ± 9.6	61.5 ± 10.2	< 0.001
< 75 years	2795 (90.8)	715 (98.5)	2080 (88.5)	
≥ 75 years	282 (9.2)	11 (1.5)	271 (11.5)	
Sex				< 0.001
Female	522 (17.0)	74 (10.2)	448 (19.1)	
Male	2555 (83.0)	652 (89.8)	1903 (80.9)	
Body weight, kg	70.0 ± 11.0	71.9 ± 10.8	69.5 ± 11.0	< 0.001
BMI, kg/m^2^	25.2 ± 3.1	25.4 ± 3.1	25.2 ± 3.1	0.136
*Prasugrel*				
5 mg	1203 (39.10)	103 (14.19)	1100 (46.79)	< 0.0001
10 mg	1874 (60.90)	623 (85.81)	1251 (53.21)	
*Clinical characteristics*				
Hypertension	1636 (53.2)	334 (46.0)	1302 (55.5)	< 0.001
Dyslipidemia	1052 (34.2)	185 (25.5)	867 (36.9)	< 0.001
Diabetes mellitus	906 (29.4)	182 (25.1)	724 (30.8)	0.003
Chronic kidney disease	48 (1.6)	8 (1.1)	40 (1.7)	0.255
Heart failure	70 (2.3)	7 (1.0)	63 (2.7)	0.007
Smoking status				< 0.001
Never-smoker	1235 (40.1)	265 (36.5)	970 (41.3)	
Ex-smoker	606 (19.7)	131 (18.0)	475 (20.2)	
Current smoker	1180 (38.4)	324 (44.6)	856 (36.4)	
Previous MI	131 (4.3)	25 (3.4)	106 (4.5)	0.214
Previous PCI	333 (10.8)	40 (5.5)	293 (12.5)	< 0.001
Previous CABG	9 (0.3)	2 (0.3)	7 (0.3)	1.000
Family history of CAD	297 (9.7)	91 (12.5)	206 (8.8)	0.003
Clinical presentation				< 0.001
STEMI	776 (25.2)	228 (31.4)	548 (23.3)	
NSTEMI	746 (24.2)	192 (26.5)	554 (23.6)	
Unstable angina	1555 (50.5)	306 (42.2)	1249 (53.1)	
*Angiographic characteristics*				
Diseased vessels				0.001
One vessel disease	1493 (48.5)	401 (55.2)	1092 (46.5)	
Two vessel disease	948 (30.8)	202 (27.8)	746 (31.7)	
Three vessel disease	635 (20.6)	123 (16.9)	512 (21.8)	
Treated lesion				0.822
Left main coronary artery	54/3804 (1.4)	10/876 (1.1)	44/2928 (1.5)	
Left anterior descending artery	1818/3804 (47.8)	425/876 (48.5)	1393/2928 (47.6)	
Left circumflex artery	792/3804 (20.8)	183/876 (20.9)	609/2928 (20.8)	
Right coronary artery	1131/3804 (29.7)	257/876 (29.3)	874/2928 (29.9)	
ACC/AHA lesion type				< 0.001
Type A	369/3557 (10.4)	169/830 (20.4)	200/2727 (7.3)	
Type B1	913/3557 (25.7)	280/830 (33.7)	633/2727 (23.2)	
Type B2	658/3557 (18.5)	162/830 (19.5)	496/2727 (18.2)	
Type C	1617/3557 (45.5)	219/830 (26.4)	1398/2727 (51.3)	
*Procedural characteristics*				
IVUS usage	1084/3075 (35.3)	245/726 (33.8)	839/2349 (35.7)	0.331
DES usage	2812/2907 (96.7)	696/726 (95.9)	2116/2181 (97.0)	0.131
Procedure success	2159/2259 (95.6)	463/482 (96.1)	1696/1777 (95.4)	0.560
Lesion success	1985/2087 (95.1)	454/473 (96.0)	1531/1614 (94.9)	0.318
Device success	2003/2101 (95.3)	457/476 (96.0)	1546/1625 (95.1)	0.429

Numbers represent mean ± standard deviation for continuous variables or number of patients (percentage) for categorical variables. BMI = Body mass index; CAD = coronary artery disease; PCI = percutaneous coronary intervention; CABG = coronary artery bypass grafting; DES = drug-eluting stent; IVUS = intravascular ultrasound-guided; STEMI = ST-segment elevation myocardial infarction; NSTEMI = non-ST-segment elevation myocardial infarction

Regarding prasugrel use in the switch cohort, the mean time elapsed since index PCI to prasugrel initiation was 15.1 ± 30.9 days; the distribution of this parameter is shown in Additional file 1: Fig. S1. As shown in Table 2, the most common reason for conversion to prasugrel in the switch cohort was the necessity for a more potent antiplatelet agent (switching from clopidogrel to prasugrel; 56.3%) followed by decreased medication compliance with a twice-daily regimen of the previous agent (switching from ticagrelor to prasugrel; 27.7%) (Table 2). The reason for switching to a more potent antiplatelet agent was a clinical decision made by the primary physician, rather than an assessment based on a laboratory test (i.e. platelet function test or genetic test). The median follow-up duration of the total cohort was 341 days (IQR 292, 365 days), while this was shorter in the switch cohort compared to the naïve cohort (339 days [IQR 286, 364 days] vs. 346 days [IQR 309, 368 days], *p* = 0.0160).

**Table 2:** Reasons for Switching to Prasugrel*

	Switch cohort
	N (%) (Total N = 2351)
Necessity for a more potent antiplatelet agent	1324 (56.3)
Decreased medication compliance with a twice-daily regimen	652 (27.7)
Adverse events of the previous agent	247 (10.5)
Drug interaction between the previous agent and other concomitant medications	91 (3.9)
Over-inhibition of platelet aggregation of the previous agent	37 (1.6)

*The reason for switching to a more potent antiplatelet agent was a clinical decision made by the primary physician

### Incidence of major cardiac and cerebrovascular events

**Fig. 3:**
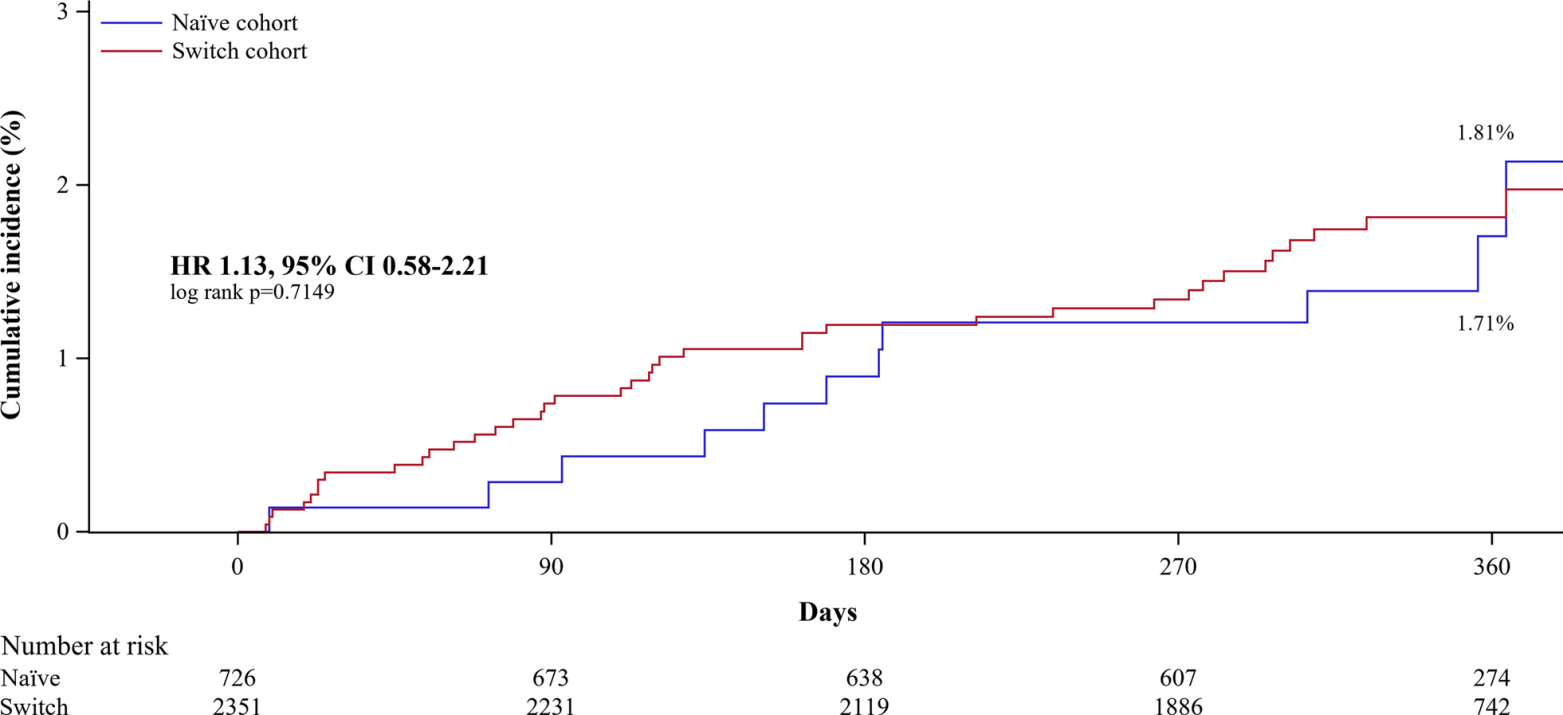
Kaplan–Meier curves for the incidence of NACE

During a 12-month follow-up period, 51 patients (1.7%) experienced at least one NACE. The incidence of NACE did not differ between the naïve and switch cohort (1.5% vs. 1.7%, *P* = 0.677). In multivariate analysis, the switch cohort was not associated with a higher risk of NACE (HR 1.17, 95% CI 0.56–2.43, *P* = 0.677, Additional file 1: Table S1), while STEMI was associated with a higher risk of NACE (HR 2.02, 95% CI 1.11–3.66, *P* = 0.021, Additional file 1: Table S1). A Kaplan-Meir survival analysis showed a similar result (log-rank *P* = 0.715, Fig. 3).

### Secondary endpoints

**Fig. 4:**
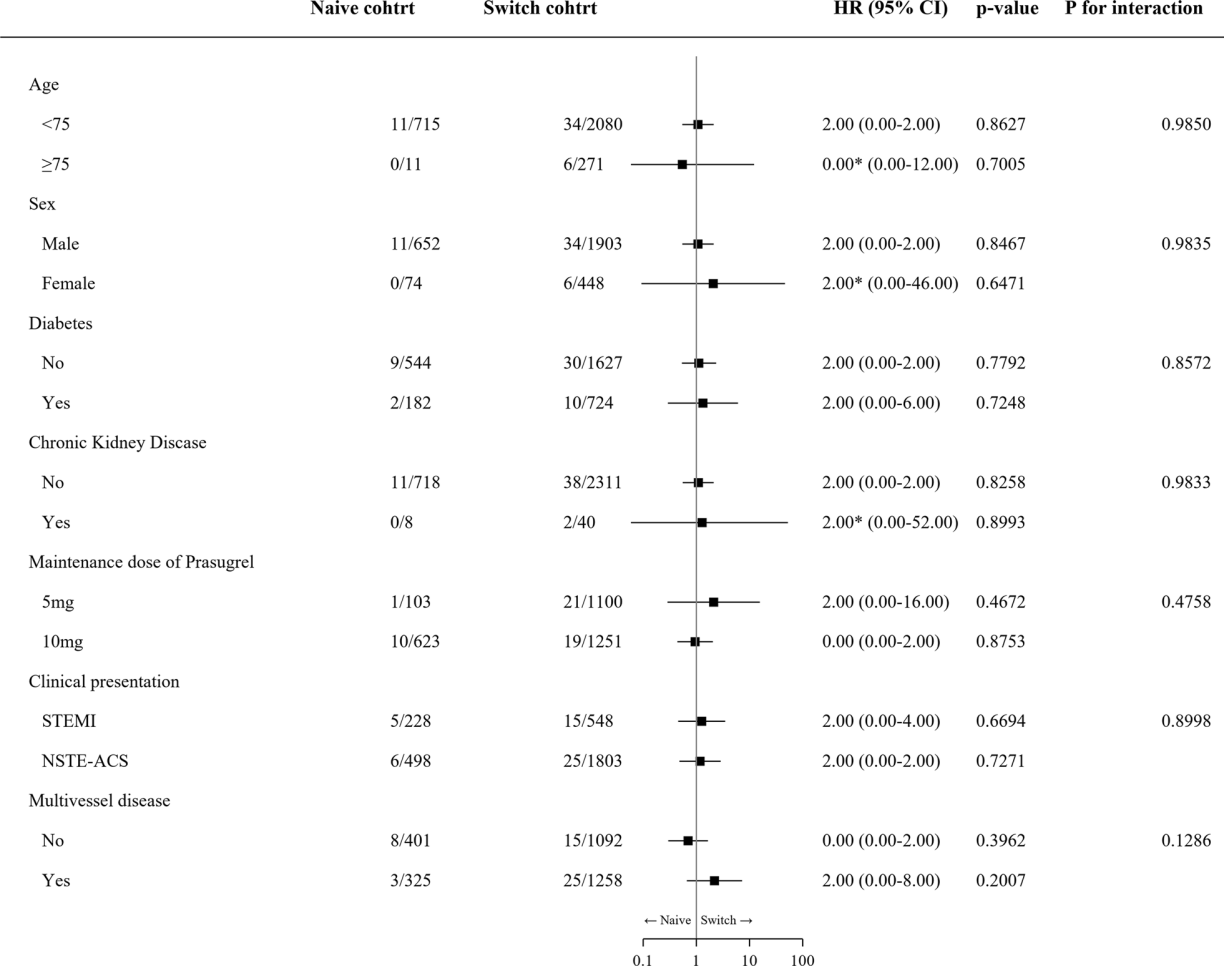
Subgroup analysis of the incidence of NACE. CKD = Chronic kidney disease; STEMI = ST-segment elevation myocardial infarction; NSTE-ACS = non-ST-segment elevation acute coronary syndrome

Table 3 presents the results for different composite endpoints and the incidence rates for each individual event. No significant differences in the effectiveness endpoint and safety endpoint were found between the naïve and switch cohort. The occurrence rates for other individual endpoints were also similar in both cohorts. In multivariate analysis, the treatment strategy (naïve and switch cohort) was not associated with a higher risk of key secondary endpoints, while old age (≥ 75 years old) (HR 2.06, 95% CI 1.04–4.07, *P* = 0.037, Additional file 1: Table S2) and STEMI (HR 2.76, 95% CI 1.78–4.28, *P* < 0.001, Additional file 1: Table S2) were associated with a higher risk of the safety endpoint. In subgroup analysis, no significant interaction was observed between the treatment strategy and the incidence of NACE across various subgroups (Fig. 4). Additionally, there was no difference in the adverse event rate according to the reason why prasugrel was switched from either a different agent, within the switch cohort (Additional file 1: Table S3).

**Table 3:** Clinical Endpoints

	Total (N = 3077)	Naïve cohort (N = 726)	Switch cohort (N = 2351)	HR [95% CI]	*P*-value
NACE	51 (1.7)	11 (1.5)	40 (1.7)	1.17 [0.56, 2.43]	0.677
Key secondary endpoints					
Effectiveness endpoint	27 (0.9)	4 (0.6)	23 (1.0)	1.96 [0.56, 6.86]	0.291
Safety endpoint	88 (2.9)	26 (3.6)	62 (2.6)	0.71 [0.43, 1.17]	0.180
*Individual events*					
All-cause death	14 (0.5)	2 (0.3)	12 (0.5)	0.89 [0.17, 4.59]	0.886
Cardiovascular death	2 (0.1)	1 (0.1)	1 (0.04)	0.03 [0.00, 3.03]	0.138
Nonfatal MI	14 (0.5)	3 (0.4)	11 (0.5)	1.68 [0.35, 8.12]	0.517
Nonfatal stroke	11 (0.4)	0 (0)	11 (0.5)	NA [0.0, NA]	0.990
Stent thrombosis	10 (0.3)	0 (0)	10 (0.4)	NA [0.0, NA]	0.991
Target vessel revascularization	15 (0.5)	4 (0.6)	11 (0.5)	0.59 [0.17, 2.08]	0.416
Bleeding					
TIMI major bleeding	27 (0.9)	7 (1.0)	20 (0.9)	0.91 [0.36, 2.28]	0.836
TIMI minor bleeding	72 (2.3)	21 (2.9)	51 (2.2)	0.72 [0.42, 1.24]	0.237

A multivariable cox regression analysis was performed by including the variables with a *P* < 0.1. Effectiveness endpoint denotes a composite of cardiovascular death, nonfatal MI, and nonfatal stroke; Safety endpoints denotes a composite of TIMI major or minor bleeding unrelated to CABG

### Adverse events

During a 12-month follow-up period, 399 events reported in 345 patients (11.2%) were classified as adverse drug reactions (ADRs). The common ADRs, with a frequency of 1% or higher, included contusion (3.9%), epistaxis (1.8%), and increased tendency to bruise (1.2%). A total of 48 events reported in 44 patients (1.4%) were classified as serious ADRs. Serious ADRs with a frequency of 0.1% or higher included cardiac disorders (0.3%), vascular stent stenosis (0.2%), coronary artery stenosis (0.2%), gastric ulcer hemorrhage (0.1%), and hematochezia (0.1%). In 182 patients (5.9%), prasugrel was discontinued due to adverse events, such as contusion (1.0%), epistaxis (0.7%), and other events occurring at rates lower than 0.5%. Comparative analysis of the results for the naïve and switch cohort did not show significant differences in the rates for ADRs (10.6%, 77/726 vs. 11.4%, 268/2351, *P* = 0.554), serious ADRs (1.1%, 8/726 vs. 1.5%, 36/2351, *P* = 0.394), and AEs leading to prasugrel discontinuation (5.5%, 40/726 vs. 6.0%, 142/2351, *P* = 0.597).

## Discussion

**Fig. 5:**
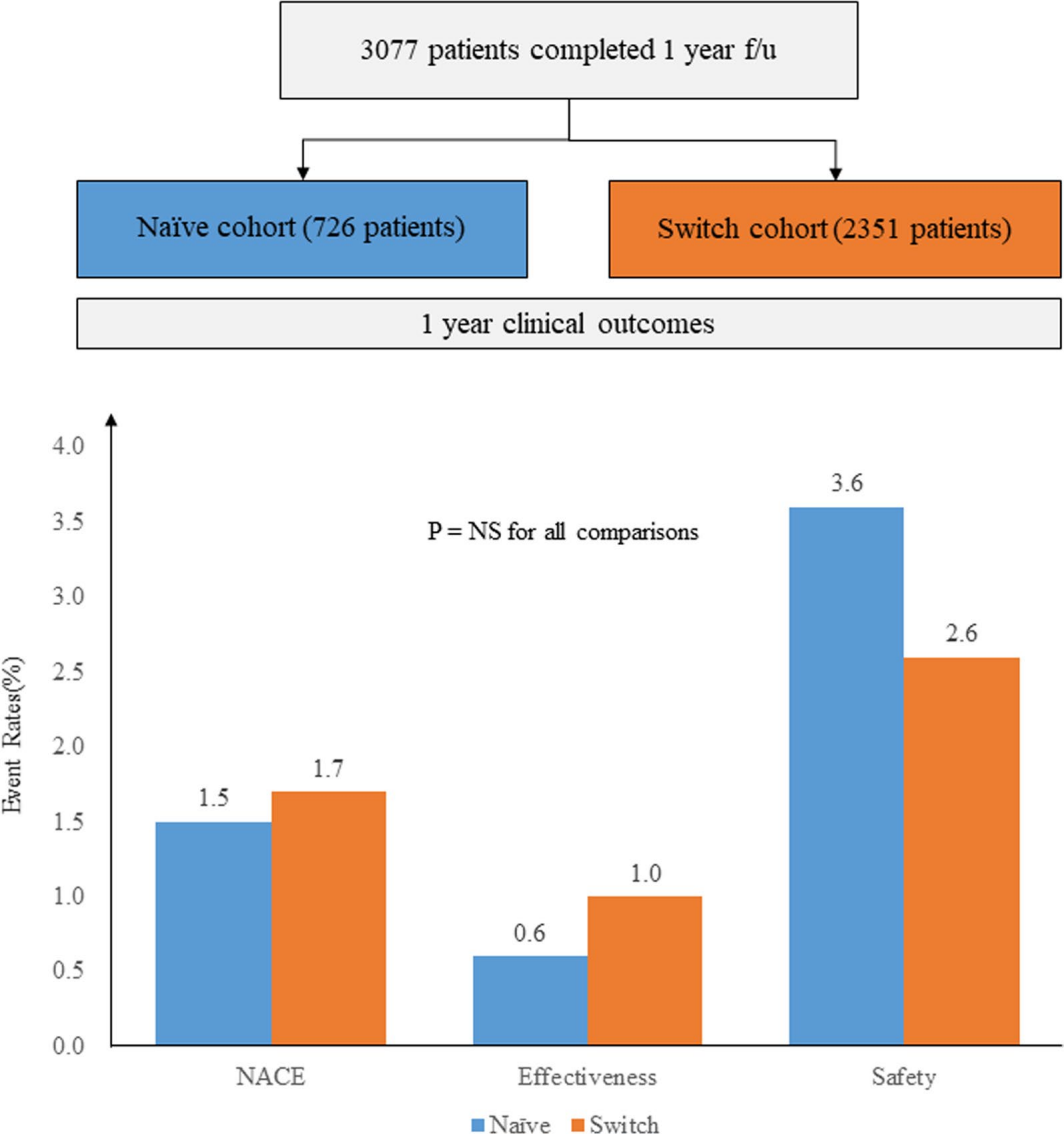
One year clinical outcomes. NACE denotes a composite of cardiovascular death, non-fatal myocardial infarction, non-fatal stroke, or TIMI major bleeding unrelated to CABG; Effectiveness endpoint denotes a composite of cardiovascular death, nonfatal MI, and nonfatal stroke; Safety endpoints denotes a composite of TIMI major or minor bleeding unrelated to CABG

This real-world study included 3077 ACS patients who received prasugrel therapy after PCI. Among the entire population, 726 patients were P2Y_12_ inhibitor-naïve, and the other 2,351 were prescribed prasugrel as a substitute agent after clopidogrel or ticagrelor pre-treatment (switch cohort). The incidence of NACE, defined as cardiovascular death, non-fatal MI, non-fatal stroke, or TIMI major bleeding unrelated to CABG, was 1.7% in the entire study population, with no significant difference found between the naïve and the switch cohort. Also, no significant differences were found between the cohorts in terms of the key secondary endpoints and adverse events (Fig. 5).

The study provided clinically relevant insight into the safety and effectiveness of prasugrel in Korean ACS patients who are potential candidates for treatment with this potent P2Y_12_ inhibitor. In the pivotal trial of prasugrel (TRITON-TIMI 38), the primary efficacy endpoint (a composite of cardiovascular mortality, non-fatal MI, or non-fatal stroke) occurred in 9.9% and major bleeding in 2.4% of patients who received this agent [5]. Meanwhile, in a recent Korean PMS study which included 3283 patients with ACS who underwent successful PCI, the efficacy outcome (a composite of cardiovascular death, MI, stroke, stent thrombosis or unplanned coronary revascularization) occurred in 0.85% and major bleeding events in 0.93% [14]. The event rate in our study was markedly lower than that of the pivotal trial [5], but similar as in the recent PMS study of Korean patients [14]. According to the authors of the latter study, the lower incidence of the composite endpoint defined as cardiovascular death, MI, stroke, stent thrombosis and unplanned CABG might be attributed to selective characteristics of the patients, with a lesser representation of those with established risk factors of bleeding, as well as to the progress in the strut design and drug coating of stents [15]. Additionally, in the switch cohort, the clinical events did not include those which occurred the initial period after PCI. Because a majority of adverse events, both ischemic and bleeding, occur in this period, excluding this phase may explain the low event rate of our population.

According to the literature, the decision to switch from one antiplatelet agent to another may be driven by various factors, including clinical setting, patient characteristics, concomitant therapies, costs, social issues, development of side effects, medication adherence, and patient/physician preference [16]. However, it needs to be stressed that although switching between P2Y_12_ receptor-inhibiting therapies has been practiced increasingly nowadays, it has not been systematized in any published guidelines, and most evidence and recommendations in this matter originate from pharmacodynamic and registry data [6, 17–19]. Our present study identified a number of reasons to switch to prasugrel. The most common cause was the necessity for a more potent antiplatelet agent (56.3%), resulting in a change from clopidogrel to prasugrel. The second most common reason (27.7%) was an intent to increase the medication compliance through switching from a twice-daily regimen (ticagrelor) to a once-daily regimen (prasugrel). The third cause was the occurrence of adverse events after the previously administered drug (10.5%). Such a distribution of the reasons to switch reflects the strengths of prasugrel as a potent P2Y_12_ inhibitor with a low adverse event rate and the once-daily regimen that promotes higher medication compliance. Regarding the clinical outcomes, they appeared to be similar in the naïve and switch cohort, even though the latter included patients with more clinical and procedural risk factors. Overall, prasugrel was well-tolerated and equally efficacious in all patients, even if not used as a primary treatment.

The results of the present study should be discussed in the context of the East Asian paradox. Based on the observation that East Asian patients are less prone to thrombotic events and more prone to bleeding, it has been suggested that their threshold of platelet reactivity is different than in Caucasians [20]. While this notion was confirmed in the case of clopidogrel [11], the results for the potent P2Y_12_ inhibitors in the East Asian population are inconclusive, with anti-ischemic benefits outweighing the risk of bleeding in some [14, 21–23] albeit not all studies [9, 24]. In addition, in patients with ACS managed invasively, in-hospital decreases in hemoglobin levels ≥ 3 g/dl, even in the absence of overt bleeding events, were common and independently associated with an increased risk for all-cause mortality at 1 year in accordance with the MATRIX trial (PMID: 33509394) [25]. As a result, many physicians in South Korea are still reluctant to apply the Western guidelines for antiplatelet agent use [10]. However, the results of the present study, as well as the outcomes of the recent PMS study [14], suggest that prasugrel can be used safely in Korean ACS patients after PCI.

### Study limitations

The primary limitation of this study stems from the lack of a control group. Furthermore, no robust statistical analyses could be conducted given the small size of some subgroups. Additionally, the actual event rate was lower than the expected numbers that were used for sample size calculation. This may be partially explained by the fact in the switch cohort, the clinical events which occurred the initial period after PCI were not included in analysis. While these limitations should be considered during the interpretation of the results, also the strengths of the study related to its real-world character should be highlighted, namely, large sample size and access to information on atypical prescription patterns.

## Conclusions

DAPT with prasugrel seems to be safe and effective both as a primary treatment and as a substitute for other P2Y_12_ inhibitors in the routine management of Korean ACS patients after PCI.

## Supplementary Information

**Additional file 1: Figure S1** Distribution of duration from index PCI to prasugrel initiation in switch cohort. **Table S1** Multivariate analysis for NACE. **Table S2** Multivariate analysis for secondary endpoints. **Table S3** Adverse event rate within the switch cohort according to the reason of prasugrel substitution. **Table S4** The list of Institutional Review Board.

## Data Availability

The datasets generated and/or analysed during the current study are available from the corresponding author on reasonable request.

## Abbreviations

ACS: Acute coronary syndrome
MI: Myocardial infarction
PCI: Percutaneous coronary intervention
NACE: Net adverse clinical event
TIMI: Thrombolysis in myocardial infarction
CABG: Coronary-artery bypass grafting
