# Supplementary material for: Real-world evidence of switching P2Y12 receptor–inhibiting therapies to prasugrel after PCI in patients with ACS: results from EFF-K registry
Source: BMC Cardiovasc Disord. 2023 Jan 9;23:6. doi: 10.1186/s12872-022-03034-5 (PMC9827633; doi:10.1186/s12872-022-03034-5)
Supplement: Supplementary file 1 — Additional file 1: Figure S1 Distribution of duration from index PCI to prasugrel initiation in switch cohort. Table S1 Multivariate analysis for NACE. Table S2 Multivariate analysis for secondary endpoints. Table S3 Adverse event rate within the switch cohort according to the reason of prasugrel substitution. Table S4 The list of Institutional Review Board. [file 12872_2022_3034_MOESM1_ESM.docx]

**Supplementary Information**

**Figure S1.** Distribution of duration from index PCI to prasugrel initiation in switch cohort

**
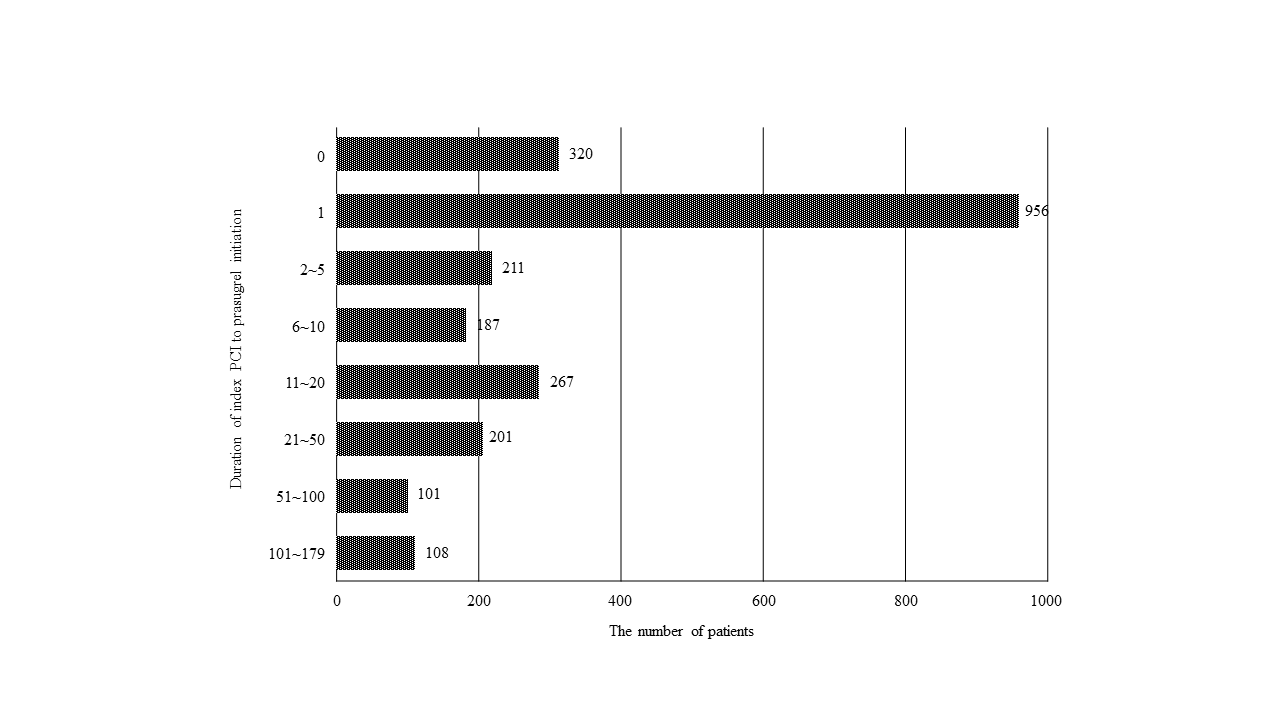
**

**Table S1.** Multivariate analysis for NACE

|  | **HR** | **95%CI** | **P-Value** |
| --- | --- | --- | --- |
| **Age**  ≥ 75 years vs < 75 years (reference) | 1.56 | (0.61, 3.95) | 0.350 |
| **Sex**  Female vs Male (reference) | 0.63 | (0.25, 1.58) | 0.330 |
| **Prasugrel dose**  5mg vs 10mg (reference) | 1.28 | (0.70, 2.37) | 0.424 |
| **DM**  DM vs Non-DM (reference) | 0.70 | (0.35, 1.38) | 0.301 |
| **Hypertension**  HTN vs Non-HTN (reference) | 1.15 | (0.64, 2.05) | 0.647 |
| **Smoke**  Current smoker vs Non-current smoker (reference) | 1.03 | (0.56, 1.89) | 0.935 |
| **Clinical presentation**  STEMI vs Non (reference) | 2.02 | (1.11, 3.66) | 0.021 |
| **Multivessel Disease**  MVD(2+3VD) vs 1VD (reference) | 1.30 | (0.73, 2.30) | 0.376 |
| **Cohort**  Switch vs Naïve (reference) | 1.17 | (0.56, 2.43) | 0.677 |

**Table S2.** Multivariate analysis for secondary endpoints

| **Endpoints** |  | **HR** | **95%CI** | **P-Value** |
| --- | --- | --- | --- | --- |
| **Effectiveness endpoints** | **Age**  ≥ 75 years vs < 75 years (reference) | 1.12 | (0.31, 4.10) | 0.861 |
|  | **Sex**  Female vs Male (reference) | 0.55 | (0.15, 1.99) | 0.366 |
|  | **Prasugrel dose**  5mg vs 10mg (reference) | 1.72 | (0.76, 3.92) | 0.196 |
|  | **DM**  DM vs Non-DM (reference) | 0.60 | (0.24, 1.51) | 0.278 |
|  | **Hypertension**  HTN vs Non-HTN (reference) | 2.53 | (1.04, 6.15) | 0.040 |
|  | **Smoke**  Current smoker vs Non-current smoker (reference) | 1.34 | (0.58, 3.09) | 0.490 |
|  | **Clinical presentation**  STEMI vs Non (reference) | 1.45 | (0.62, 3.42) | 0.394 |
|  | **Multivessel Disease**  MVD(2+3VD) vs 1VD (reference) | 1.68 | (0.75, 3.81) | 0.210 |
|  | **Cohort**  Switch vs Naïve (reference) | 1.96 | (0.56, 6.86) | 0.291 |
| **Safety endpoints** | **Age**  ≥ 75 years vs < 75 years (reference) | 2.06 | (1.04, 4.07) | 0.037 |
|  | **Sex**  Female vs Male (reference) | 0.62 | (0.31, 1.22) | 0.163 |
|  | **Prasugrel dose**  5mg vs 10mg (reference) | 0.99 | (0.61, 1.60) | 0.952 |
|  | **DM**  DM vs Non-DM (reference) | 1.36 | (0.87, 2.13) | 0.181 |
|  | **Hypertension**  HTN vs Non-HTN (reference) | 1.38 | (0.88, 2.16) | 0.161 |
|  | **Smoke**  Current smoker vs Non-current smoker (reference) | 0.81 | (0.51, 1.28) | 0.358 |
|  | **Clinical presentation**  STEMI vs Non (reference) | 2.76 | (1.78, 4.28) | <0.001 |
|  | **Multivessel Disease**  MVD(2+3VD) vs 1VD (reference) | 1.16 | (0.76, 1.78) | 0.496 |
|  | **Cohort**  Switch vs Naïve (reference) | 0.71 | (0.43, 1.17) | 0.180 |

**Table S3.** Adverse event rate within the switch cohort according to the reason of prasugrel substitution

|  | Necessity for a more potent antiplatelet agent  (N=1324) | Decreased medication compliance with a twice-daily regimen  (N=652) | Adverse events of the previous agent  (N=247) | Drug interaction between the previous agent and other concomitant medications  (N=91) | | Over-inhibition of platelet aggregation of the previous agent  (N=37) | P-Value |
| --- | --- | --- | --- | --- | --- | --- | --- |
| NACE | 32 (2.4) | 9 (1.4) | 1 (0.4) | | 1 (1.1) | 0 | 0.172 |
| Key Secondary Endpoints |  |  |  | |  |  |  |
| - Effectiveness endpoint | 20 (1.5) | 4 (0.6) | 1 (0.4) | | 0 | 0 | 0.317 |
| - Safety endpoint | 37 (2.8) | 22 (3.4) | 10 (4.1) | | 1 (1.1) | 1 (2.7) | 0.634 |
| *Individual Events* |  |  |  | |  |  |  |
| - All-cause death | 7 (0.5) | 5 (0.8) | 2 (0.8) | | 0 | 1 (2.7) | 0.367 |
| - Cardiovascular death | 2 (0.2) | 0 | 0 | | 0 | 0 | 1.0000 |
| - Nonfatal MI | 9 (0.7) | 2 (0.3) | 0 | | 0 | 0 | 0.622 |
| - Stent thrombosis | 6 (0.5) | 1 (0.2) | 2 (0.8) | | 1 (1.1) | 1 (2.7) | 0.079 |
| - Urgent target vessel revascularization | 11 (0.8) | 0 | 0 | | 0 | 1 (2.7) | 0.023 |
| - Bleeding |  |  |  | |  |  |  |
| TIMI major bleeding | 16 (1.2) | 5 (0.8) | 0 | | 1 (1.1) | 0 | 0.385 |
| TIMI minor bleeding | 28 (2.1) | 20 (3.1) | 10 (4.1) | | 0 | 1 (2.7) | 0.170 |

Effectiveness endpoint denotes a composite of cardiovascular death, nonfatal MI, and nonfatal stroke; Safety endpoints denotes a composite of TIMI major or minor bleeding unrelated to CABG.

**Table S4.** The list of Institutional Review Board

| **NO** | **Institution** | **Investigator** | **IRB No.** |
| --- | --- | --- | --- |
| 1 | Seoul National University Hospital | Hyo-Soo Kim | H-1702-054-832 |
|  | 101, Daehak-ro, Jongno-gu, Seoul, 03080, Republic of Korea |  |  |
| 2 | Severance Hospital | Jung-Sun Kim | 4-2017-0075 |
|  | 50-1, Yonsei-ro, Seodaemun-gu, Seoul, 03722, Republic of Korea |  |  |
| 3 | Ulsan University Hospital | Gyung-Min Park | 2016-12-012 |
|  | 877, Banggeojinsuhnwando-ro, Dong-gu, Ulsan, 44033, Republic of Korea |  |  |
| 4 | Dongkang hospital | Hyung-Jun Kim | 42705 |
|  | 123-3, Taewha-dong, Jung-gu, Ulsan Metropolitan City, 681-711, Republic of Korea |  |  |
| 5 | Kangwon National University Hospital | Bong-Ki Lee | 2017-01-009 |
|  | 156, Baengnyeong-ro, Chuncheon-si, Gangwon-do, 24289, Republic of Korea |  |  |
| 6 | Yonsei University Wonju Severance Christian Hospital | Sung-Gyun Ahn  Jun-Won Lee | 2016-12-0037  2016-12-0038 |
|  | 20, Ilsan- ro, Wonju, Gangwon-do, 26426, Republic of Korea |  |  |
| 7 | The Catholic University of Korea, Yeouido St. Mary`s Hospital | Yun-Seok Choi | SC170SME0006 |
|  | 10,63(yuksam)-ro, Yeongdeungpo-gu, Seoul, 07345, Republic of Korea |  |  |
| 8 | Gachon University Gil Medical Center | Kyoung-Hoon Lee | CGIRB 2017-156 |
|  | 21, Namdong-daero 774 beon-gil, Namdong-gu, Incheon, 21565, Republic of Korea |  |  |
| 9 | The Catholic University of Korea Bucheon St. Mary's Hospital | Hee-Yeol Kim | HC17OSME0007 |
|  | 327, Sosa-ro, Bucheon-si, Gyeonggi-do, Republic of Korea |  |  |
| 10 | SoonChunHyang University Hospital Cheonan | Sang-Ho Park | 2017-01-002 |
|  | 31, Suncheonhyang 6-gil, Dongnam-gu, Cheonan-si, Chungcheongnam-do, Republic of Korea |  |  |
| 11 | Kangbuk Samsung Hospital | Jong-Young Lee | 2017-02-007 |
|  | 29, Saemunan-ro, Jongno-gu, Seoul, Republic of Korea |  |  |
| 12 | Inha University Hospital | Sang-Don Park | 2017-01-008 |
|  | 27, Inhang-ro, Jung-gu, Incheon, 22332, Republic of Korea |  |  |
| 13 | Hallym University Dongtan Sacred Heart Hospital | Myung-Soo Park | 2017-018-S |
|  | 7, Keunjaebong-gil, Hwaseong-si, Gyeonggi-do, 18450, Republic of Korea |  |  |
| 14 | Bongseng Memorial Hospital | Woo-Hyung Bae | BSIRB-2017-003 |
|  | 401, Jungang-daero, Dong-gu, Busan, 48775, Republic of Korea |  |  |
| 15 | Soonchunhyang University Hospital Bucheon | Yun-Hang Cho | 2017-03-014 |
|  | 170 Jomaru-ro, Wonmi-gu, Bucheon-si, Gyeonggi-do, 14584, Republic of Korea |  |  |
| 16 | Hallym University Kangnam Sacred Heart Hospital | Jung-Rae Cho | 2017-02-011 |
|  | 1, Singil-ro, Yeongdeungpo-gu, Seoul 07441, Republic of Korea |  |  |
| 17 | Kyung Hee University Hospital at Gangdong | Jin-Man Cho | 2017-01-027 |
|  | 892, Dongnam-ro, Gangdong-gu, Seoul, 05278, Republic of Korea |  |  |
| 18 | Korea University Anam Hospital | Jae-hyoung Park | 2017AN0104 |
|  | 73, Goryeodae-ro, Seongbuk-gu, Seoul, 02841, Republic of Korea |  |  |
| 19 | Ajou University Hospital | Hong-Seok Lim | AJIRB-MED-OBS-16-512 |
|  | 164, World cup-ro, Yeongtong-gu, Suwon-si, Gyeonggi-do,16499, Republic of Korea |  |  |
| 20 | Dong-A University Hospital | Kyung-Il Park | DAUHIRB-17-026 |
|  | 26, Daesingongwon-ro, Seo-gu, Busan, 49201, Republic of Korea |  |  |
| 21 | Hanyang University Medical Center | Young-Hyo Lim | 2017-01-042 |
|  | 222-1, Wangsimni-ro, Seongdong-gu, Seoul, 04763, Republic of Korea |  |  |
| 22 | Hanyang University Guri Hospital | Jeong-Hoon Shin | 2017-04-008 |
|  | 153, Gyeongchun-ro, Guri-si, Gyeonggi-do, 11923, Republic of Korea |  |  |
| 23 | Kosin University Gospel Hospital | Jung-Ho Heo | 2017-01-019 |
|  | 262, Gamcheon-ro, Seo-gu, Busan, Republic of Korea |  |  |
| 24 | Seoul National University Boramae Medical Center | Sang-Hyun Kim | 16-2017-11 |
|  | 20, Boramae-ro 5-gil, Dongjak-gu, Seoul, Republic of Korea |  |  |
| 25 | Pusan National University Yangsan Hospital | Kook-Jin Chun | 02-2017-004 |
|  | Geumo-ro 20, Mulgeum-eup, Yangsan-si, Gyeongnam, 50612, Republic of Korea |  |  |
| 26 | The Catholic University of Korea, St. Vincent’s Hospital | Su-Nam Lee | VC17OSME0029 |
|  | 93, Jungbu-daero, Paldal-gu, Suwon-si, Gyeonggi-do, Republic of Korea |  |  |
| 27 | Gangnam Severance Hospital | Young-Won Yoon | 3-2017-0016 |
|  | 211, Eonju-ro, Gangnam-gu, Seoul, Republic of Korea |  |  |
| 28 | Jeonbuk National University Hospital | Jei-Keon Chae | 2017-01-027 |
|  | 20, Geonji-ro, Deokjin-gu, Jeonju-si, Jeollabuk-do, Republic of Korea |  |  |
| 29 | The Catholic University of Korea, Uijeongbu St. Mary's Hospital | Chan-Joon Kim | UC17OSME0019 |
|  | 271, Cheonbo-ro, Uijeongbu-si, Gyeonggi-do, Republic of Korea |  |  |
| 30 | Cheonju St. Mary's Hospital | Yong-Mo Yang | IRB-118 |
|  | 173-19, Juseong-ro, Cheongwon-gu, Cheongju-si, Chungcheongbuk-do, Republic of Korea |  |  |
| 31 | Changwon Fatima Hospital | Yang-Chun Han | 44244 |
|  | 45, Changi-daero, Uichang-gu, Changwon-si, Gyeongsangnam-do, Republic of Korea |  |  |
| 32 | Daegu Catholic University Medical Center | Seung-Pyo Hong | CR-17-042-L |
|  | 33, Duryugongwon-ro 17-gil, Nam-gu, Daegu, 42472, Republic of Korea |  |  |
| 33 | Hallym University Sacred Heart Hospital | Woo-Jung Park  Sang-Ho Jo | 2017-S025  2017-S021 |
|  | 22 Gwanpyeong-ro 170beon-gil, Dongan-gu, Anyang-si, Gyeonggi-do, 431-070, Republic of Korea |  |  |
| 34 | Dongguk University Ilsan Hospital | Ji-Hyeon Kim | 2017-24 |
|  | 27, Dongguk-ro, Ilsandong-gu, Goyang-si, Gyeonggi-do, Republic of Korea |  |  |
| 35 | Chung-Ang University Hospital | Wang-Soo Lee | 1760-001-283 |
|  | 102, Heukseok-ro, Dongjak-gu, Seoul, Republic of Korea |  |  |
| 36 | Sungae Hospital | Bae-Keun Kim | SA2017-02 |
|  | 22 Yoidaebang-ro 53 Road, Yondeungpo-gu, Seoul, Republic of Korea |  |  |
| 37 | Sejong Hospital | Chi-Hoon Kim | 2017-338 |
|  | 28, Hohyeon-ro 489beon-gil, Bucheon-si, Gyeonggi-do, Republic of Korea |  |  |
| 38 | Pusan National University Hospital | Kwang-Soo Cha | D-1703-006-063 |
|  | 179, Gudeok-ro, Seo-gu, Busan, Republic of Korea |  |  |
| 39 | Ewha Womans University Medical Center | Kyung-Jin Kim Ki-Hwan Kwon | EUMC-2017-03-027  EUMC-2017-04-039 |
|  | 1071, Anyangcheon-ro, Yangcheon-gu, Seoul 07985, Republic of Korea |  |  |
| 40 | Maryknoll Hospital | Hyeon-Gook Lee | MMC/2017-239 |
|  | 121, Junggu-ro, Jung-gu, Busan, Republic of Korea |  |  |
| 41 | KyungHee University Medical Center | Won Kim | 2017-05-057 |
|  | 23, Kyung Hee Dae-ro, Dongdaemun-gu, Seoul 02447, Republic of Korea |  |  |
| 42 | Chonnam National University Hospital | Young-Joon Hong | CNUH-2017-139 |
|  | 42, Jebong-ro, Dong-gu, Gwangju, Republic of Korea |  |  |
| 43 | The Catholic University of Korea, Seoul St. Mary’s Hospital | Hun-Jun Park | KC17OODE0266 |
|  | 222, Banpo-daero, Seocho-gu, Seoul, Republic of Korea |  |  |
| 44 | Inje University Haeundae Paik Hospital | Dong-ki Kim | 2017-07-015 |
|  | 875, Haeundaero, Haeundae-gu, Busan, 612-896, Republic of Korea |  |  |
| 45 | Samsung Changwon Hospital | Yong-Hwan Park | 2017-07-003 |
|  | 158, Paryong-ro, MasanHoewon-gu, Changwon-si, Gyeongsangnsm-do, 51353, Republic of Korea |  |  |
| 46 | National Health Insurance Service Ilsan Hospital | Sung-Jin Oh | 2017-09-003 |
|  | 100, Ilsan-ro, Ilsandong-gu, Goyang-si, Gyeonggi-do, Republic of Korea |  |  |
| 47 | Carollo General Hospital | Min-Goo Lee | SCH2017-0106 |
|  | 221, Sungwang-ro, Suncheon-si, Jeollanam-do, 57931, Republic of Korea |  |  |
| 48 | Seoul National University Bundang Hospital | Jung-Won Suh | B-1801/442-404 |
|  | 82, Gumi-ro 173beon-gil, Bundang-gu, Seongnam-si, Gyeonggi-do, 13620, Republic of Korea |  |  |
| 49 | Daejeon St. Mary's Hospital | Man-Won Park | DC18OODE0004 |
|  | Daejeon St. Mary's Hospital. 64, Daeheung-ro, Jung-gu, Daejeon, Republic of Korea |  |  |
| 50 | Korea University Guro Hospital | Seung-Woon Rha  Cheol-Ung Choi | 2018GR0015  2017GR0008 |
|  | 148, Gurodong-ro, Guro-gu, Seoul, Republic of Korea |  |  |
| 51 | Hallim Hospital | Jeong-Min Bong | 2017-006 |
|  | 722, Jangje-ro, Gyeyang-gu, Incheon, Republic of Korea |  |  |
| 52 | Hallym University Chuncheon Sacred Heart Hospital | Sang-Min Park | 2018-25 |
|  | 14, Gwanpyeong-ro 176beon-gil, Dongan-gu, Anyang-si, Gyeonggi-do, Republic of Korea |  |  |
